# The *Pseudomonas aeruginosa* Autoinducer 3O-C12 Homoserine Lactone Provokes Hyperinflammatory Responses from Cystic Fibrosis Airway Epithelial Cells

**DOI:** 10.1371/journal.pone.0016246

**Published:** 2011-01-31

**Authors:** Matthew L. Mayer, Jared A. Sheridan, Christoph J. Blohmke, Stuart E. Turvey, Robert E. W. Hancock

**Affiliations:** 1 Department of Microbiology and Immunology, University of British Columbia, Vancouver, Canada; 2 Centre for Microbial Diseases and Immunity Research, University of British Columbia, Vancouver, Canada; 3 Department of Pediatrics, University of British Columbia, Vancouver, Canada; 4 Child and Family Research Institute, BC Children's Hospital, Vancouver, Canada; Centre de Recherche Public de la Santé (CRP-Santé), Luxembourg

## Abstract

The discovery of novel antiinflammatory targets to treat inflammation in the cystic fibrosis (CF) lung stands to benefit patient populations suffering with this disease. The *Pseudomonas aeruginosa* quorum sensing autoinducer N-3-oxododecanoyl homoserine lactone (3O-C12) is an important bacterial virulence factor that has been reported to induce proinflammatory cytokine production from a variety of cell types. The goal of this study was to examine the ability of 3O-C12 to induce proinflammatory cytokine production in normal and CF bronchial epithelial cells, and better understand the cellular mechanisms by which this cytokine induction occurs. 3O-C12 was found to induce higher levels of IL-6 production in the CF cell lines IB3-1 and CuFi, compared to their corresponding control cell lines C38 and NuLi. Systems biology and network analysis revealed a high predominance of over-represented innate immune pathways bridged together by calcium-dependant transcription factors governing the transcriptional responses of A549 airway cells to stimulation with 3O-C12. Using calcium-flux assays, 3O-C12 was found to induce larger and more sustained increases in intracellular calcium in IB3-1 cells compared to C38, and blocking this calcium flux with BAPTA-AM reduced the production of IL-6 by IB3-1 to the levels produced by C38. These data suggest that 3O-C12 induces proinflammatory cytokine production in airway epithelial cells in a calcium-dependent manner, and that dysregulated calcium storage or signalling in CF cells results in an increased production of proinflammatory cytokines.

## Introduction

Cystic fibrosis (CF) is a chronic pulmonary disease characterized by recurrent and excessive inflammation that causes the destruction of lung tissue, eventually resulting in respiratory failure. Inflammation in the CF lung is thought to be driven by both host factors such as cytokines [1]–[3] and bacterial factors [4]–[6] produced during lung colonization with pathogens such as *Pseudomonas aeruginosa*. *P. aeruginosa* relies on quorum sensing molecules such as the autoinducer N-3-oxododecanoyl homoserine lactone (3O-C12) to drive the expression of numerous genes related to virulence [7], biofilm formation [8], and antibiotic resistance [9] when colonizing the CF lung. Studies in mice have shown that 3O-C12 is a critical determinant for bacterial fitness and establishing chronic lung infections [10], [11], leading many to argue that the development of quorum sensing inhibitors would be a major advance in the ability to eradicate infections with *P. aeruginosa* [12]–[14].

In addition to the role played by 3O-C12 in regulating bacterial virulence, this molecule has recently been reported to have numerous immunomodulatory and inflammatory properties. 3O-C12 has a powerful inhibitory effect on professional immune cells, inhibiting dendritic cell and T-cell activation [15], promoting apoptosis [16]–[18] and inhibiting the ability of macrophage and monocytes to respond to a range of Toll-like receptor (TLR) agonists through disruption of NF-κB signalling [19]. Perplexingly, 3O-C12 is a powerful inducer of proinflammatory cytokines such as IL-6 and IL-8 in airway epithelial cells and lung fibroblasts, upregulates inflammatory enzymes such as cyclooxygenase-2 (COX-2) (both *in vitro* and *in vivo*) [10], [20]–[22], and enhances neutrophil chemotaxis [23]. Taken together, these data suggest that 3O-C12 suppresses the function of key immune networks responsible for bacterial clearance, while simultaneously enhancing inflammatory pathways that promote the pathogenesis of CF.

Cells from CF patients exhibit an exaggerated inflammatory response to stimulation with *P. aeruginosa* [24]–[27], and we have previously identified interactions between flagellin and TLR5 as contributing to this phenotype using CF airway epithelial cell lines and primary CF peripheral blood mononuclear cells *in vitro* [4] and in a recent CF patient cohort candidate SNP analysis study [28]. One limitation of these previous studies was the use of heat-killed bacteria or laboratory strains with diminished virulence to control for motility or cytotoxicity differences between different *P. aeruginosa* strains and mutants, an experimental approach that precluded our ability to identify factors secreted by virulent live cells, such as 3O-C12. In this study, we report that 3O-C12 also differentially induces the increased production of the proinflammatory cytokine IL-6 in CF airway epithelial cells. Using systems biology and network analysis approaches, we demonstrated that the inflammatory response of airway epithelial cells to 3O-C12 relied on calcium-dependent transcription factors, and identify exaggerated calcium signalling in CF airway epithelial cells as the mechanism underlying their exaggerated cytokine response to 3O-C12.

## Results

### Homoserine lactone 3O-C12 elicited markedly higher IL-6 production from CF airway epithelial cells

**Figure 1:**
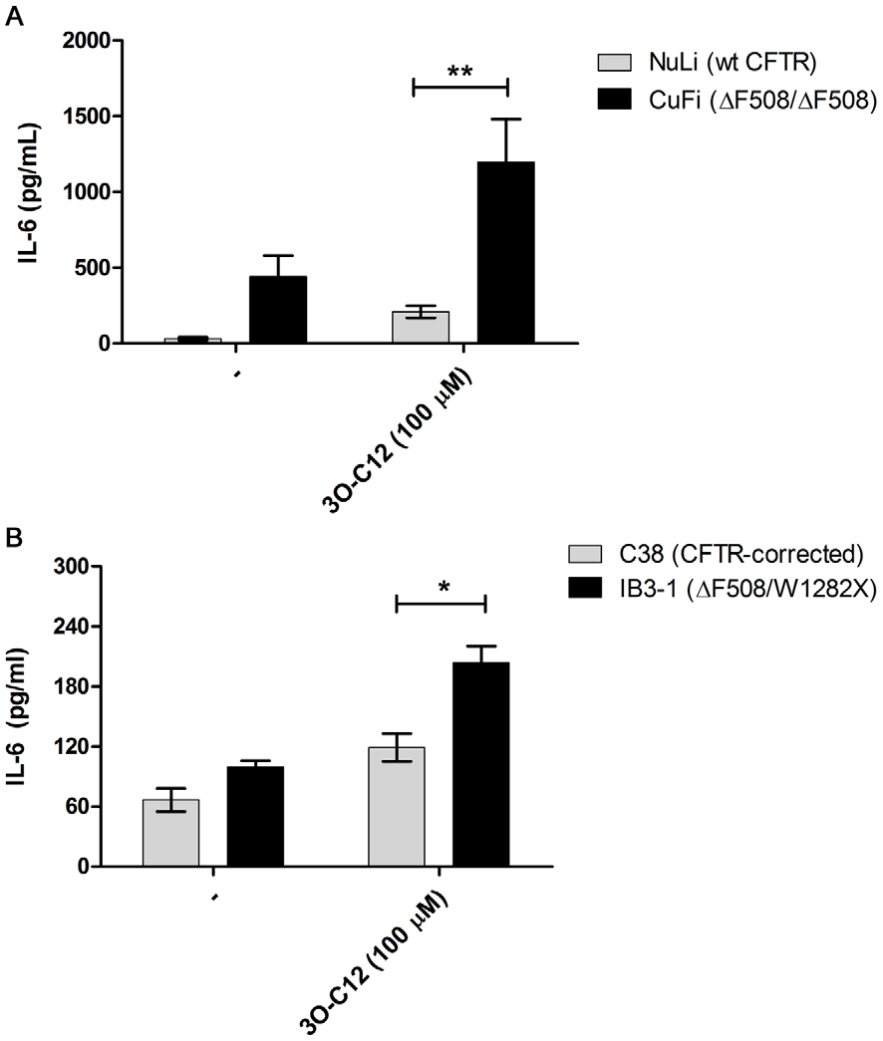
CF airway cells produce more IL-6 in response to 3O-C12. Cytokine production was measured in supernatants from (A) CuFi and (B) IB3-1, or their matched non-CF counterparts 24 hr after treatment with 3O-C12 (100 µM). Bars show the mean of three independent experiments ± SEM; * p<0.05; ** p<0.01.

To examine *P. aeruginosa* quorum sensing autoinducers for their ability to induce exaggerated proinflammatory responses in CFs airway epithelial cells, two pairs of matched CF and non-CF airway epithelial cells (NuLi and CuFi; C38 and IB3-1) were stimulated with 3O-C12 and C4 homoserine lactones at concentrations between 10 and 100 µM. This range was selected to be consistent with the concentration of 3O-C12 used to elicit immunomodulatory or inflammatory effects in both *in vitro* cell models [20], [21], [23], [29], [30], [15], [31], [32], [17], [16], and *in vivo* animal models [10]. When tested at 100 µM, 3O-C12 was found to induce the proinflammatory cytokine IL-6 in all four cell types tested, however the induction of IL-6 was between 2 and 4-fold greater in the CF cell lines CuFi and IB3-1 compared to their non-CF matched counterparts NuLi and C38 (Figure 1A and 1B). The difference in cytokine induction was not due to differences in cytotoxicity, as cell viability for all four cell lines was consistently >95% after 24 hr stimulation with concentrations of 3O-C12 between 10 and 100 µM (compared to 250 µM which induced cell death in 18–27% of cells) as determined by LDH release. Interestingly, 3O-C12 was highly specific for the induction of IL-6, as stimulation of cells with up to 100 µM of 3O-C12 did not elicit the production of the proinflammatory cytokine IL-8. To confirm that the absence of IL-8 did not arise from cell specific defects in the synthesis of this cytokine, cells were stimulated with flagellin (0.1 µg/ml) or IL-1β (10 ng/ml) for 24 hours. Flagellin and IL-1β induced the production of both IL-6 and IL-8 from all four cells with IL-8 production being on the order of 1 to 2-fold higher than IL-6 (data not shown), confirming that 3O-C12 was specific in its ability to induce IL-6. C4 HSL did not induce the production of IL-6 or IL-8 to greater than basal levels in any of the four cell lines tested.

### Systems biology and network analysis of airway cell inflammatory responses to 3O-C12 implicating innate immune pathways and calcium signalling

Having identified 3O-C12 as capable of inducing greater IL-6 production from CF airway cells, we sought to understand the mechanism by which this phenomenon occurred. The signalling pathways that govern cell responses to 3O-C12 are poorly elucidated; the molecule modulates the activity of peroxisome proliferator-activated receptor-gamma (PPARγ) [31] but no specific receptor(s) and/or signalling pathway(s) have been identified as mediators of 3O-C12 cytokine induction. To gain further insights into the mechanisms by which 3O-C12 induces proinflammatory cytokine secretion in airway epithelial cells, we employed systems biology and network based analysis of a recent microarray dataset which examined the transcriptional responses of A549 airway cells to this molecule. A total of 2,989 genes were identified by Bryan *et al*. [33] as differentially expressed (DE) after 6 hr stimulation with 3O-C12 (50 µM), and these genes were uploaded to InnateDB so that over-representation analysis could be carried out.

A total of 30 pathways were found to be significantly over-represented by the DE genes, of which a substantial number (21/30) related to the activation of cellular innate immune and inflammatory responses (Table 1). These included *TNF-alpha pathway* (p = 0.0007), *NOD-like receptor signaling pathway* (p = 0.0017), *canonical NF-kappaB pathway* (p = 0.0276) *MAPK signaling pathway* (p = 0.0416), and *JNK cascade* (p = 0.0427). Similarly, significantly over-represented gene ontology (GO) functional terms (Table 2, Table S1) included *innate immune response* (p = 0.0001), *inflammatory response* (p = 0.0017), *cytokine production* (p = 0.0175), and *positive regulation of I-kappaB kinase/NF-kappaB cascade* (p = 0.0303). A second theme present within the over-representation analysis results was presence of calcium sensitive transcription factors such as the *ATF-2 transcription factor network* (p = 0.0006), *calcineurin-regulated NFAT-dependent transcription* (p = 0.0027; includes NFATC1 and NFATC2), and *Calcium signaling in the CD4^+^ TCR pathway* (p = 0.0407). Similarly, additional significant GO terms included *metal ion binding* (p = 3.58×10^-7^), *response to calcium ion* (p = 0.0185), *calcineurin complex* (p = 0.0189), and *cation transmembrane transporter activity* (p = 0.0305).

**Table 1:** Pathway over-representation analysis of differentially expressed (DE) genes in A549 cells stimulated with 3O-C12 HSL.

Pathway Name^1^	Number of genes		Genes Ratio	p-value^2^
	In pathway	DE in dataset		
Gene expression of IL2 by AP-1	5	5	100%	0.0007
Activation of Chaperones by IRE1alpha	4	3	75%	0.0428
Extrinsic prothrombin activation pathway	13	6	46%	0.0074
Mets affect on macrophage differentiation	18	8	44%	0.0020
TRAF6 Mediated Induction of the antiviral cytokine IFN-alpha/beta cascade	18	7	39%	0.0081
Role of mitochondria in apoptotic signaling	13	5	38%	0.0431
Intrinsic prothrombin activation pathway	23	8	35%	0.0069
Toll Like Receptor 3 (TLR3) Cascade	21	7	33%	0.0180
JNK cascade	19	6	32%	0.0427
Canonical NF-kappaB pathway	23	7	30%	0.0276
ATF-2 transcription factor network	49	14	29%	0.0006
Calcium signaling in the CD4+ TCR pathway	25	7	28%	0.0407
Calcineurin-regulated NFAT-dependent transcription in lymphocytes	44	12	27%	0.0027
CD40/CD40L signaling	30	8	27%	0.0285
NOD-like receptor signaling pathway	62	15	24%	0.0017
IL12-mediated signaling events	59	14	24%	0.0028
IL4	51	12	24%	0.0077
IL6-mediated signaling events	45	11	24%	0.0091
P53 signaling pathway	68	15	22%	0.0033
Regulation of Androgen receptor activity	51	11	22%	0.0238
Glucocorticoid receptor regulatory network	80	17	21%	0.0024
Direct p53 effectors	135	26	19%	0.0007
NOTCH	79	15	19%	0.0121
Small cell lung cancer	84	15	18%	0.0218
TGF-beta signaling pathway	86	15	17%	0.0257
Regulation of nuclear SMAD2/3 signaling	82	14	17%	0.0430
T cell receptor signaling pathway	83	14	17%	0.0433
TNFalpha	189	31	16%	0.0008
Pathways in cancer	325	41	13%	0.0045
MAPK signaling pathway	270	32	12%	0.0416

1. InnateDB pathway over-representation analysis tool; http://www.innatedb.ca.

2. Benjamini-Hochberg corrected p-value for multiple comparisons.

**Table 2:** Gene ontology (GO) term over-representation analysis of differentially expressed (DE) genes in A549 cells stimulated with 3O-C12 HSL showing select^1^ terms pertaining to innate immunity and calcium signalling.

GO term [ontology domain]	Number of genes		Genes Ratio	p-value^2^
	In GO term	DE in dataset		
calcineurin complex [cellular component]	4	2	50%	0.0189
MAP kinase tyrosine/serine/threonine phosphatase activity [molecular function]	13	6	46%	4.69E-05
cytokine production [biological process]	10	3	30%	0.0175
response to calcium ion [biological process]	27	5	19%	0.0185
cation transmembrane transporter activity [molecular function]	41	6	15%	0.0305
inflammatory response [biological process]	252	27	11%	0.0017
positive regulation of I-kappaB kinase/NF-kappaB cascade [biological process]	112	12	11%	0.0303
innate immune response [biological process]	606	58	10%	0.0001
cytokine activity [molecular function]	191	19	10%	0.0163
transcription factor activity [molecular function]	1050	98	9%	2.39E-06
metal ion binding [molecular function]	2485	202	8%	3.58E-07

1. Complete GO term over-representation analysis results can be found in Table S1.

2. Benjamini-Hochberg corrected p-value for multiple comparisons.

**Figure 2:**
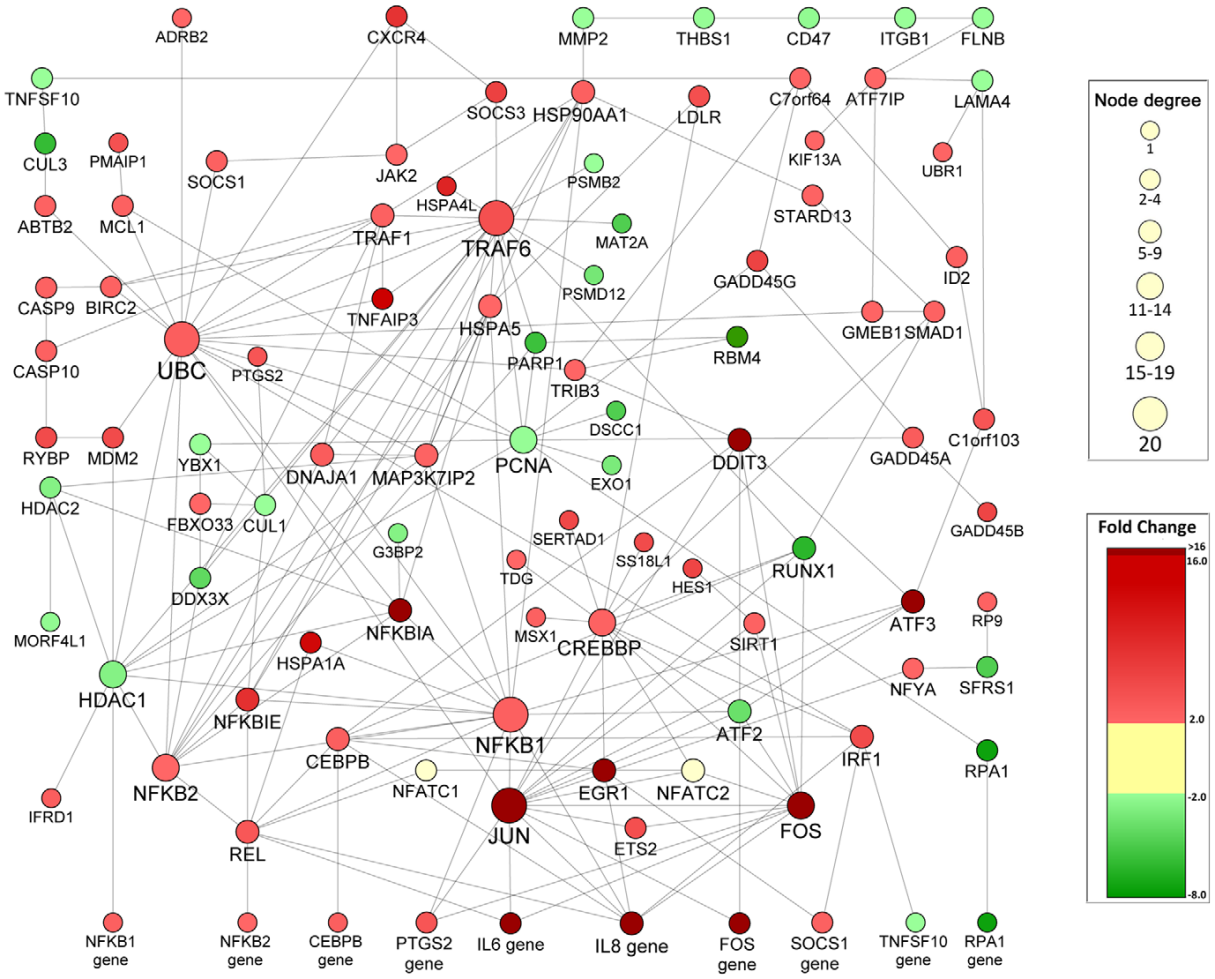
Network analysis of transcriptional responses of A549 cells to 3O-C12 showing protein-protein interactions. The color of gene nodes is proportional to their relative fold-change versus untreated cells, node-node connections represent known protein-level interactions annotated within InnateDB, and the node size reflects its degree of interconnectivity (hub-like nature) within the network.

Although the calcium dependent transcription factors ATF-2, NFATC1, and NFATC2 were identified by InnateDB's pathway ORA, the latter two genes were not themselves differentially expressed. This was not an unexpected result, as many key regulatory elements of signalling pathways in mammalian cells are regulated by non-transcriptional controls [34], [35], and so do not appear as DE genes within microarray datasets. To ensure that the NFAT transcription factors were included in our analysis using an unbiased approach, we resubmitted a gene list to InnateDB containing the 2,989 genes, and then visualized protein level interactions between genes using Cytoscape and the software plugin Cerebral. This analysis yielding a network containing 361 gene nodes with 773 unique protein-protein or protein-gene interactions (Figure S1A). In addition, InnateDB was tasked to create a first-order interaction network for NFATC1 and NFATC2 (Figure S1B), and the two resulting networks were merged into a single entity (Figure S1C) containing 396 gene nodes and 866 unique protein-protein or protein-gene interactions. To control for biases which might have arisen by intentionally introducing two non-DE genes into the dataset, jActive was used to identify the single most significant subnetwork that contributes to the manner in which A549 cells responded to HSL. Using this method, a single highly statistically significant subnetwork containing 101 nodes (including NFATC1 and NFATC2) with 198 unique interactions was found (Figure 2), although different cell types might express individual nodes to different extents.

Analysis of this critical subnetwork revealed that numerous key innate immune signalling pathways, including the MAP Kinase, JAK, and canonical NF-κB pathways, converging in the upregulation of IL-6. In addition, network analysis also revealed that different innate immune pathways were linked together by calcium-activated transcription factors. The calcium-dependant TF NFATC1 provided a bridging point between the NF-κB family members C-Rel (REL) and p52 (NFKB2) and EGR1. Furthermore, the network analysis suggested the likelihood that NFATC2 integrated signalling between EGR1, CREB, and AP-1 (Fos/Jun) in the upregulation of the proinflammatory cytokine IL-6 in A549 cells. Based on these findings, we hypothesized that 3O-C12 mediated induction of proinflammatory cytokines in airway epithelial cells was largely mediated by calcium-dependent processes and transcription factors.

### 3O-C12 induces increased levels of [Ca^2+^]_i_ in the CF cell line IB3-1 that is in turn drives increased cytokine production

**Figure 3:**
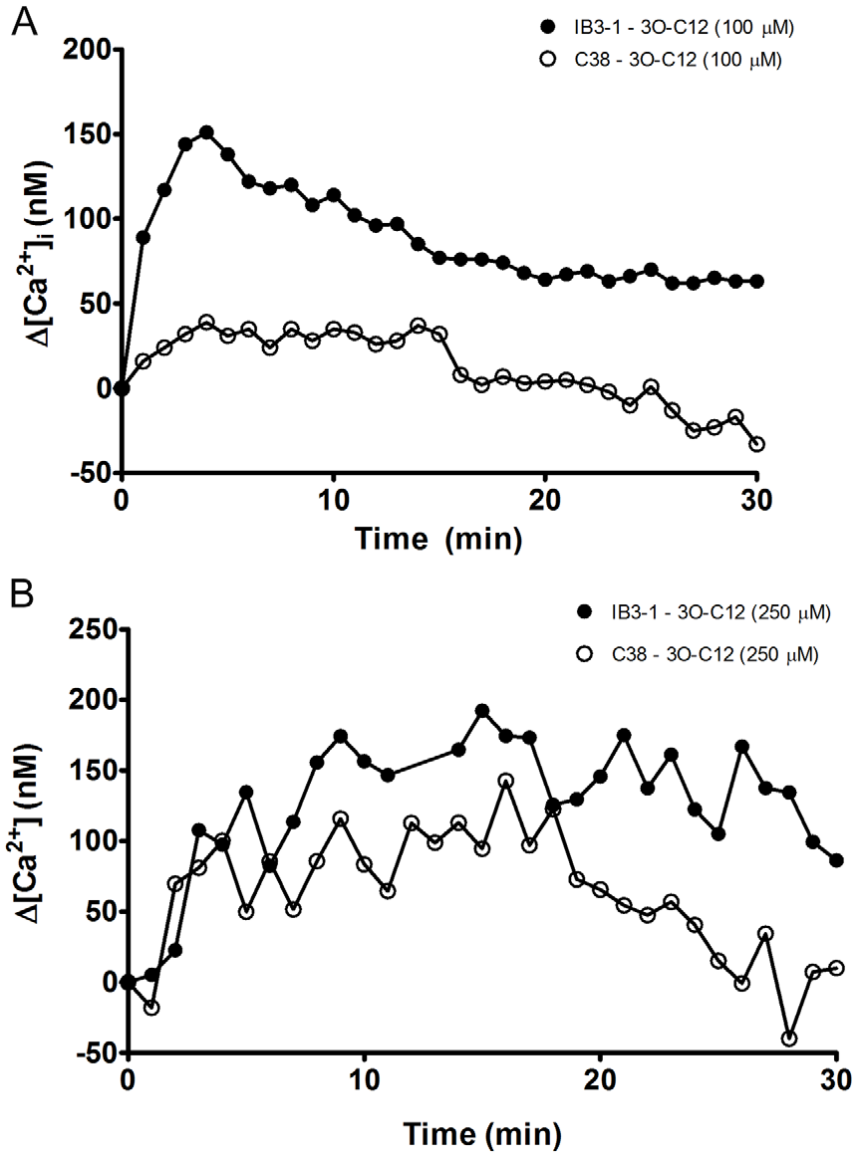
3O-C12 triggers larger and more sustained rises in intracellular calcium in IB3-1 cells. CF cell line IB3-1 and the isogenic CFTR-corrected cell line C38 were stimulated with 3O-C12 at (A) 100 µM or (B) 250 µM. Data are reported as Δ[Ca^2+^]**_I_**, the change in [Ca^2+^]**_i_** in stimulated cells minus the change in vehicle treated cells. Data points show the mean of 6 replicate wells with serial measurements made every 50–60 s over 30 min. Graph is representative of one of three replicate experiments with similar results.

We next sought to confirm these *in silico* findings using calcium-flux assays to determine if stimulation of IB3-1 and C38 with 3O-C12 induced differences in the levels of intracellular Ca^2+^ using a fluor-4 microplate based assay system to examine kinetic changes in the second. Concentrations of HSL ranging from 10 to 50 µM failed to induce substantial increases in intracellular calcium in C38 and IB3-1 (data not shown), whereas at concentrations over 100 µM, Δ[Ca^2+^]_i_ rose significantly within minutes for the CFTR defective line IB3-1, but not the corrected line C38 (Figure 3A). At a higher concentration (250 µM) of 3O-C12, similar increases in Δ[Ca^2+^]_i_ were observed in both cell lines, however [Ca^2+^]_i_ returned to basal levels in C38 by the 20 min mark but remained elevated in IB3-1 for the duration of the time course (Figure 3B). Although 250 µM 3O-C12 was found to be cytotoxic, it is unlikely that cytotoxicity contributes to the differences in [Ca^2+^]_i_ between IB3-1 and C38. Shiner *et al*. (2006) [36] reported that cell death following 3O-C12 stimulation is due to sustained increases in intracellular Ca^2+^, therefore the change in [Ca^2+^]_i_ that occurred over the short (30 min) time frame used in this assay most certainly precede any LDH release detected after 24 hr.

**Figure 4:**
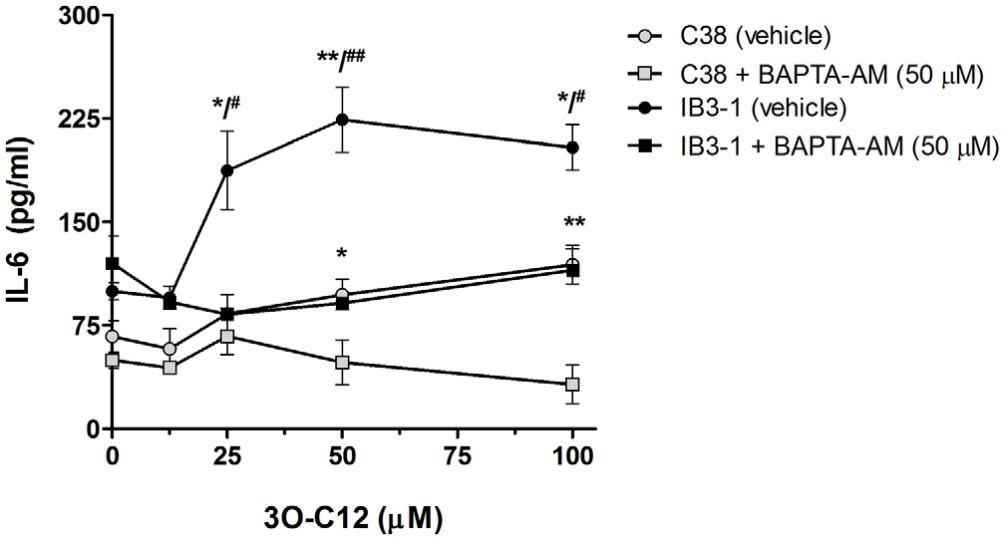
Heightened induction of IL-6 in IB3-1 by 3O-C12 is antagonized by inhibiting intracellular calcium. Cells were pre-treated for 1 hr with vehicle or BAPTA-AM (intracellular calcium chelator). Data points are the mean of three independent experiments ± SEM. Statistical significance for comparisons between vehicle and BAPTA-AM within a cell line are indicated with *; comparisons between IB3-1 (vehicle) and C38 (vehicle) are indicated with #; */# p<0.05; **/## p<0.01.

To investigate if the 3O-C12 induced elevations and sustained increases in [Ca^2+^]_i_ in IB3 were responsible for differential induction of proinflammatory cytokines in these two cell types, cells were stimulated with increasing doses of 3O-C12, with or without a 1-hr pretreatment with membrane-permeable calcium chelator BAPTA-AM. Over a range of HSL concentrations, BAPTA-AM antagonized the exaggerated production of IL-6 by IB3-1 cells, reducing the levels to those produced by C38 (Figure 4).

## Discussion

Colonization of the CF lung by *P. aeruginosa* induces robust neutrophilic inflammation that is disproportionate to the bacterial load. Ongoing inflammation results in pulmonary obstruction, reduced lung function and ultimately respiratory failure and death [37]. In the present study, we demonstrated that in addition to *P. aeruginosa* flagellin [24], [4], [28], 3O-C12 homoserine lactone induces a heightened production of the proinflammatory cytokine IL-6 from two different CF respiratory epithelial cell lines. The concentrations of 3O-C12 used in this study to elicit IL-6 production fell between 10 µM and 100 consistent with previous studies examining the immunomodulatory or inflammatory properties of this ligand [20], [21], [23], [29], [30], [15], [31], [32], [17], [16]. Although 3O-C12 has only been detected in the 1–20 nM range in CF patient sputum [7], and in the 1–2 µM range in murine *P. aeruginosa* lung infection models [38], *in vitro* biofilm models predict that localized pockets of 3O-C12 production exist in which concentrations of the autoinducer readily approach 600 µM [39].

The signalling pathways in mammalian cells triggered by 3O-C12 are poorly characterized, and so we employed a systems biology approach to provide a further understanding of how this molecule induces inflammatory cytokine production. Using a previously published microarray dataset [33], we discovered direct engagement and activation of multiple immune pathways by 3O-C12, with 21/30 over-represented pathways related to immune processes involved in cell activation and cytokine production (Table 1). Network analysis of the dataset carried out to identify the most significant sub-network of genes, based on their protein-level interactions, further identified that 3O-C12 induction of proinflammatory cytokines occurs in tandem with the upregulation of the classical cytokine transcription factors NF-κB, Fos, and Jun (Figure 2). Further upstream, upregulation of the CXCR4-UBC and CXCR4-SOCS3-TRAF6 pathways was apparent (Figure 2). CXCR4, which monogamously binds CXCL12 (SDF-1), is able to recruit progenitor epithelial cells to the lungs in response to acute airway injury [40] suggesting that airway specific stress-response pathways are being triggered by 3O-C12.

3O-C12 has been shown to elicit the production of IL-8 from fibroblasts and corneal epithelial cells [21], [30], and the IL-8 gene was upregulated 47-fold in the microarray analysis of A549 cells stimulated with this ligand [33], so it was unsurprising that the gene was found to be a statistically significant node in the network analysis of this data set (Figure 2). However a previous study examining the effect of soluble *Burkholderia cepacia* products on A549 cells detected IL-8 production following stimulation with bacterial supernatant, but not after stimulation with 3O-C12 [41], consistent with the absence of detectable IL-8 protein after stimulating NuLi, CuFi, IB3-1 or C38 with the homoserine lactone, but not with other proinflammatory stimuli such as flagellin. The discrepancy between IL-8 transcriptional and protein levels could be due to the fact that IL-8 mRNA undergoes extensive post-transcriptional regulation [42], and the reliance of 3O-C12 on non-canonical signalling pathways [43] is insufficient in some cell types to induce the cellular machinery required for proper processing and secretion of this cytokine.

The results of our systems biology and network analysis suggested that intracellular Ca^2+^ may also play a role in proinflammatory cytokine production by airway epithelial cells in response to 3O-C12. Intracellular Ca^2+^ is an important second messenger that has a well appreciated role in the downstream activation cellular processes with proinflammatory sequelae. The results of our systems biology analysis suggested that intracellular Ca^2+^ may be contributing to cytokine induction by 3O-C12 as multiple Ca^2+^-influenced transcription factors involved in inflammatory responses, such as c-Jun, NF-κB, and NFATc [44], were significant nodes in the network graph (Figure 2). 3O-C12 has recently been shown to trigger Ca^2+^ release from the endoplasmic reticulum in airway epithelial cells [45], an event which has been linked in other cell types to the ability of this molecule to induce apoptosis [36], disrupt epithelial cell tight junctions [46], and circumvent host defence mechanisms against *P. aeruginosa* [32], but to our knowledge, has not been associated with the induction of proinflammatory cytokines by the autoinducer. In addition to validating our *in silico* predictions, the finding that the CFTR-modified airway epithelial cell line IB3-1 responded to 3O-C12 with increased and sustained rises in [Ca^2+^]_i_ compared to the isogenic CFTR-corrected C38 cell line suggested Δ[Ca^2+^]_i_ was mediating the differential IL-6 production between these cell lines. This hypothesis was subsequently shown to be correct as the membrane-permeable calcium chelator BAPTA-AM reduced 3O-C12-mediated IL-6 production in both cell lines. These findings are consistent with a growing body of literature demonstrating that CF cells undergo expansion of their endoplasmic reticulum Ca^2+^ stores, causing dysregulation of intracellular Ca^2+^ signalling pathways which in turn, can result in enhance proinflammatory cytokine secretion [3], [47], [48].

The *lasI/lasR* quorum sensing system responsible for the production of 3O-C12 HSL has previously been identified as a potential therapeutic target in CF. Antagonizing bacterial production of 3O-C12, either by immunization [38] or through the use of small molecule inhibitors of *lasR* [49], [50], results in decreased mortality in animal models of *P. aeruginosa* lung infection. A solid understanding of the mechanisms that propagate the inflammatory process in the CF lung is necessary to develop novel therapies that limit the magnitude, duration, or frequency of pulmonary inflammation which in turn is crucial to alleviate the burden of disease in CF and improve both the duration and quality of life in affected patients. This study sheds additional light on the etiology of inflammation in the CF lung, and provides additional rationale for the further investigation of the *P. aeruginosa* quorum sensing molecule 3O-C12 HSL as a novel therapeutic target for reducing lung inflammation in CF.

## Materials and Methods

### Reagents

N-3-oxo-dodecanoyl-L-homoserine lactone (3O-C12 HSL) and N-butanoyl-L-homoserine lactone (C4 HSL) were obtained from Cayman Chemical (Ann Arbor, MI), dissolved in DMSO, stored at −20°C and used within 3 months. Flagellin (ultrapure recombinant) and interleukin-1β were obtained from Invivogen (San Diego, CA). Ionomycin and BAPTA-AM were obtained from EMD Bioscience (Gibbstown, NJ), dissolved in ddH_2_O or DMSO respectively, and stored at −20°C. EGTA was obtained from Alfa Aesar (Ward Hill, MA) and was solubilized at 0.5M in ddH_2_O by the drop-wise addition of 10M NaOH until a pH of 8.00 was achieved.

### Cell lines and growth conditions

This study utilized the well-characterized CF lung epithelial cell line IB3-1 (compound heterozygote for the ΔF508 and W1282X CFTR mutations) and CuFi-1 (homozygrous for the ΔF508 mutation) with their matched control lung epithelial cells C38 (“corrected” CF cell line derived from IB3-1) or NuLi-1 (wild type CFTR) (American Type Culture Collection) [51], [52]. IB3-1 and C38 were grown in coated flasks (100 µg/ml BSA, 30 µg/ml bovine collagen I, and 10 µg/ml human fibronectin) in LHC-8 basal medium (Invitrogen, Carlsbad, CA) supplemented with 10% (v/v) FCS, 2 mM L-glutamine, 1 mM sodium pyruvate, and 1% penicillin-streptomycin-amphotericin B solution. CuFi-1 and NuLi-1 were grown in Primaria flasks (BD, Mississauga, ON, Canada) in BEGM medium (Lonza, Walkersville, MD) supplemented with SingleQuot growth factors (Lonza). All cells were grown at 37°C in 5% CO_2_.

### Cell stimulations

NuLi-1 and CuFi-1 were seeded into 96-well Primaria plates at a density of 1.5×10^5^ cells/ml, and allowed to grow for 18–24 hr until confluent. Growth media was aspirated and replaced with fresh media containing 3O-C12 or DMSO vehicle. C38 and IB3-1 cells were seeded into 96-well plates (coated with BSA, collagen, and fibronectin as described above) at a density of 2.5×10^5^ cells/ml, and were allowed to grow for 16–20 hr before confluent. Growth media was aspirated and replaced with fresh media containing BAPTA-AM (50 µM) or DMSO vehicle for 1 hr before the addition of 3O-C12. For all four cell lines, cell-free supernatants were collected 24 hr after the addition of 3O-C12 and either frozen at −80C for later cytokine quantification, or used immediately for LDH assays.

### ELISA and LDH assays

Supernatants were assayed for IL-6 and IL-8 using Ready Set Go ELISA kits from eBioscience (San Diego, CA), according to the manufacturer's instructions. Cell cytotoxicity was analyzed through the quantification of lactate dehydrogenase (LDH) release using a commercial cytotoxicity kit (Roche Applied Science, Laval, QC, Canada) according to the manufacturer's instructions.

### Calcium assays

C38 and IB3-1 cell lines were seeded at a density of 2.5×10^5^ cells per into 96-well view plates (Perkin-Elmer, Waltham, MA, USA) coated with BSA, collagen, and fibronectin as described above. Cells were allowed to rest for 16–20 hr, growth media was removed, and the calcium indicator dye Fluo-4 NW (Invitrogen) was used to load the cells for 1 hr according to the manufacturer's instructions. After loading the cells, two minutes of baseline fluorescence were was collected, and then cells were treated with EGTA (10 mM), ionomycin (10 µM), or 3O-C12 HSL. Fluorescence data was recorded every 50–60 sec with an excitation wavelength of 485 nm and an emission wavelength of 535 nm using a Victor 3 (Perkin-Elmer). Intracellular concentration of calcium was calculated as described by Tabary *et al*. [3] using the formula: *[Ca]_i_  =  Kd x ((F – F_EGTA_)/(F_Ionomycin_ – F)* where Kd  =  345 nM [53]. Δ[Ca^2+^]_i_ was defined as the intracellular calcium concentration in stimulated cells, less the intracellular calcium concentration in cells treated with DMSO vehicle, for a given time point. Fluorescence data from each well was normalized to its own baseline fluorescence before the addition of any treatment.

### Systems biology and network analysis

Transcriptional responses of A549 cells stimulated with 3O-C12 HSL (50 µM) for 6 hr was recently examined using microarray by Bryan *et al.* [33]. A list of differentially expressed genes with corresponding fold-changes and p-values was obtained from the paper's supplemental section, and utilized for in-depth systems biology analysis. Briefly, genes were uploaded to InnateDB and pathway and gene ontology (GO) term over-representation analysis was carried out as previously described [54]. A list of protein level interactions between differentially expressed genes was obtained from InnateDB [55], as were first-order interactions for the transcription factors NFATC1 and NFATC2 (identified as important by the over-representation analysis). Network analysis was carried out as previously described [56], [57], by visualizing these interactions and then merging them into a single integrated network using Cytoscape (2.6.3 for Windows) and the software plugin Cerebral [58]. The single most statistically significant sub-network was identified within the larger interaction network using the jActiveModule plugin for Cytoscape [34], [35], and the result was again visualized in Cerebral to appreciate network directionality within the system.

### Statistics

Statistical significance was determined using independent sample t-tests for comparisons of two treatments for a given cell line, whereas 2-way ANOVA followed by Tukey's post-hoc testing was used for comparisons of multiple conditions across two cell lines. Statistical analysis for *in* vitro cell stimulations was carried out using SPSS 17.0 for Windows. Over-representation analysis was carried out using InnateDB's default methodology (hypergeometric algorithm, Benjamini-Hochberg multiple testing p-value correction) [54], [57].

## Supporting Information

Figure S1 **Sequential construction of the network graph of A549 transcriptional responses to 3O-C12.** Microarray gene expression data [33] was uploaded to InnateDB, and protein-protein interactions were visualized in Cytoscape/Cerebral (A). Interaction networks were also constructed for protein-protein interaction for NFATC1 and NFATC2 using InnateDB, and visualized in Cytoscape/Cerebral (B). These two networks were merged into a single network (C) which was then used for subnetwork analysis with the Cytoscape plugin jActive (the results of which are shown in Figure 2).
(TIF)

Table S1 **Complete Gene ontology (GO) term over-representation analysis of differentially expressed (DE) genes in A549 cells stimulated with 3O-C12 HSL.**
(XLS)
