# Supplementary figures and images for: The Pseudomonas aeruginosa Autoinducer 3O-C12 Homoserine Lactone Provokes Hyperinflammatory Responses from Cystic Fibrosis Airway Epithelial Cells
Source: PLoS One. 2011 Jan 31;6(1):e16246. doi: 10.1371/journal.pone.0016246 (PMC3031552; doi:10.1371/journal.pone.0016246)

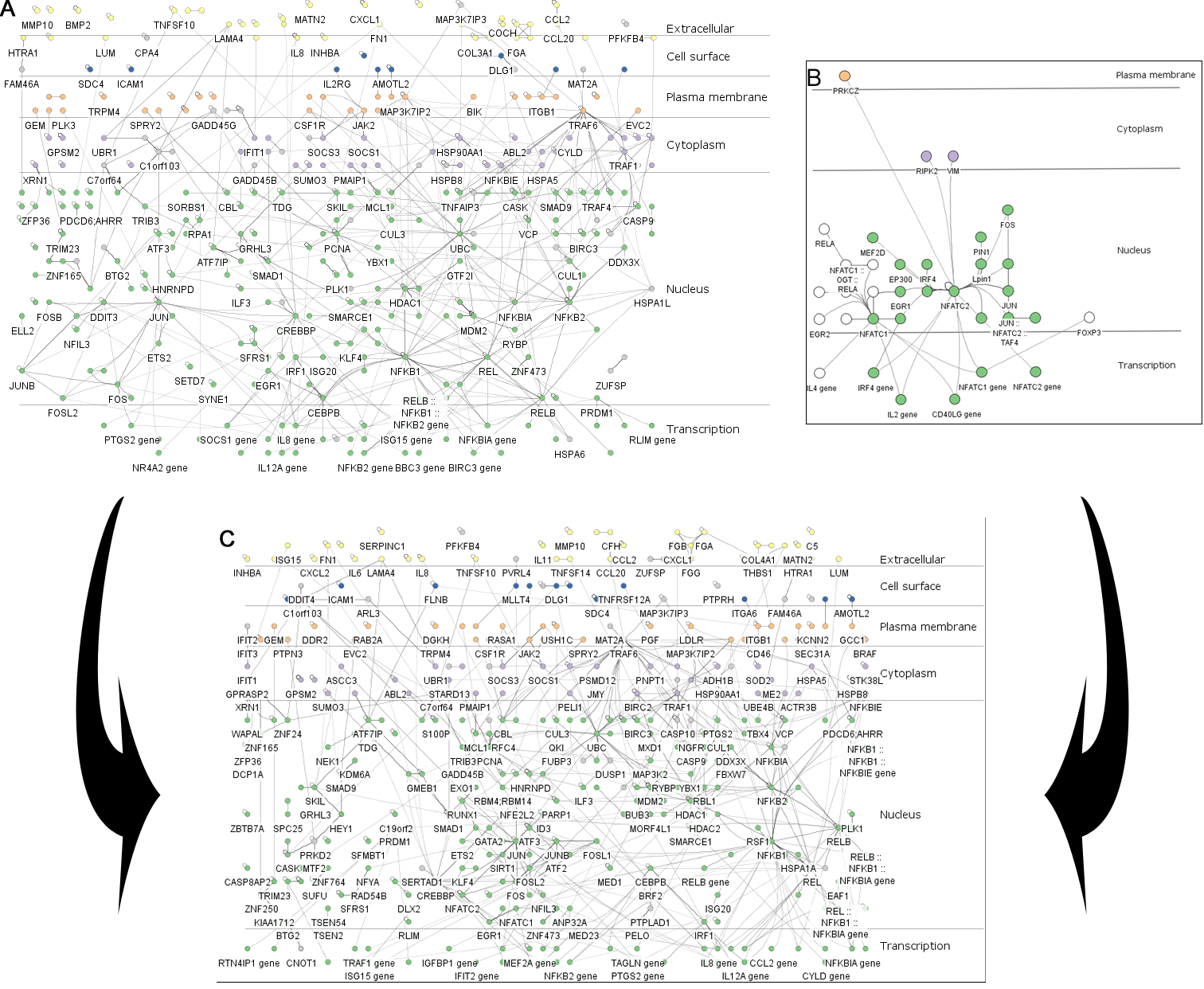

Supplement: Figure S1 — Sequential construction of the network graph of A549 transcriptional responses to 3O-C12. Microarray gene expression data [33] was uploaded to InnateDB, and protein-protein interactions were visualized in Cytoscape/Cerebral (A). Interaction networks were also constructed for protein-protein interaction for NFATC1 and NFATC2 using InnateDB, and visualized in Cytoscape/Cerebral (B). These two networks were merged into a single network (C) which was then used for subnetwork analysis with the Cytoscape plugin jActive (the results of which are shown in Figure 2). (TIF) [file pone.0016246.s001.tif]
